# Molecular docking analysis of aspirin analogues with β-catenin

**DOI:** 10.6026/97320630016725

**Published:** 2020-09-30

**Authors:** Jayaraman Selvaraj, Hussain Sardar, Veeraraghavan Vishnupriya, Janardhana Papayya Balakrishna, Surapaneni Krishna Mohan, Rajamanickam Pon Nivedha, Periyasamy Vijayalakshmi, Rajagopal Ponnulakshmi

**Affiliations:** 1 Department of Biochemistry, Saveetha Dental College and Hospitals, Saveetha Institute of Medical and Technical Sciences, Saveetha University, Chennai - 600 077, India; 2 Department of Biotechnology, Government Science College, Chitradurga-577501, Karnataka, India; 3 Department of Stem Cell Biology, Stellixir Biotech Pvt Ltd, No.V-31, 2nd floor, 10th Main Road, Peenya 2nd Stage Industrial Area, Bangalore - 560058, Karnataka, India; 4 Department of Biochemistry and Department of Clinical Skills & Simulation, Panimalar Medical College Hospital & Research Institute, Varadharajapuram, Poonamallee, Chennai - 600 123, India; 5 Exonn Biosciences, Ticel Bio Park, Chennai-600 0113, India; 6 PG & Research Department of Biotechnology & Bioinformatics, Holy Cross College (Autonomous), Trichy- 620002, Tamil Nadu, India; 7 Central Research Laboratory, Meenakshi Academy of Higher Education and Research (Deemed to be University), Chennai-600 078, India

**Keywords:** Colon cancer, Wnt signaling, molecular docking, ADME

## Abstract

Canonical Wnt signaling pathway plays a crucial role in cancer cell proliferation, which links by the growth of β-catenin in cell due to inactivation of glycogen synthetase kinase-3. Therefore, it is of interest to design novel candidates to bind with β-catenin. Hence, we document the molecular docking analysis data of aspirin analogues with β-catenin for further consideration.

## Background

Colorectal cancer (CRC) is the most common form of cancer in oncologic pathology, and it is ranked as second most recurrent cause of death associated to cancer, it affecting the both men as well as women in the same manner worldwide, developed and underdeveloped Countries. It is also predicted to overcome the death ratio of chronic diseases in the upcoming years [1]. Almost 1.8 million new cases were identified in 2018 all over the word. In case of India, it has been expected that about 1 in 23 (4.4%) for men and 1 in 25 (4.1%) for women respectively. The Wnt/β-Catenin signaling pathway plays a crucial role in the transcriptional regulation process that impacts cell growth, development, and differentiation in many malignancies, including CRC [2]. β-Catenin, activitation deregulate the Wnt proteins, so a downstream activator of the Wnt signaling pathway, have been concerned in several cancers [3]. The majority of sporadic forms of colorectal cancer having mutation in key element of the Wnt/β-Catenin signaling cascade, particularly in Adenomatous polyposis coli (APC) and β-Catenin, thereby increasing the transcriptional activity of the latter [4]. β-Catenin target genes play an ultimate role in tissue homeostasis, initiation and progression of CRC through the regulation of various cellular processes, including proliferation, stem cell fate, survival, differentiation, migration and angiogenesis [5]. Particularly, the genes involved in proliferation and migration ware over expressed in CRC [5]. Many drugs to inhibit the proliferation targeted these genes. Aspirin is one of the best-marketed drug to act against Wnt signaling pathway. It reduces the death rate of colon cancer patients [6] and also shrinks the size of colonic adenomatous polyps both in human and animal studies [7]. Aspirin have the capacity to modulate the Wnt signaling at many levels, including effector pathways of COX-2/PGE2, activity of the β-catenin destruction complex, and the expression of key Wnt target genes involved in tumorigenesis Therefore, it is of interest to design novel candidates to bind with β-catenin. The nine analogues (Table 1 - see PDF ) were used in the present study.

## Materials & Methods:

### Protein structure:

Crystal structure of Beta-catenin was retrieved from PDB ((PDB Id: 1JDH) [8] is used in this study [8].

### Ligand data:

Structure data for aspirin and its analogues were downloaded from pubchem database. All compounds were converted as PDB file format using the Online Smile Translator. Energy minimizations were done using ChemBio 3D Ultra 12.0 as per the standard method.

### Molecular docking:

Patch dock [9,10] was used for the molecular docking analysis of aspirin analogues with β catenin.

### ADME analysis of selected compounds:

The drug capability and pharmacokinetic estimation of the compounds were carried out by Lipinski filter (http://www.scfbio-iitd.res.in/software/drugdesign/lipinski.jsp), according to which an orally active drug must follow at least of four of the five laid down condition for drug likeness namely: molecular mass, cLogP, hydrogen donor and acceptor and molar refractive index [11].

## Results and Discussion:

**Figure 1:**
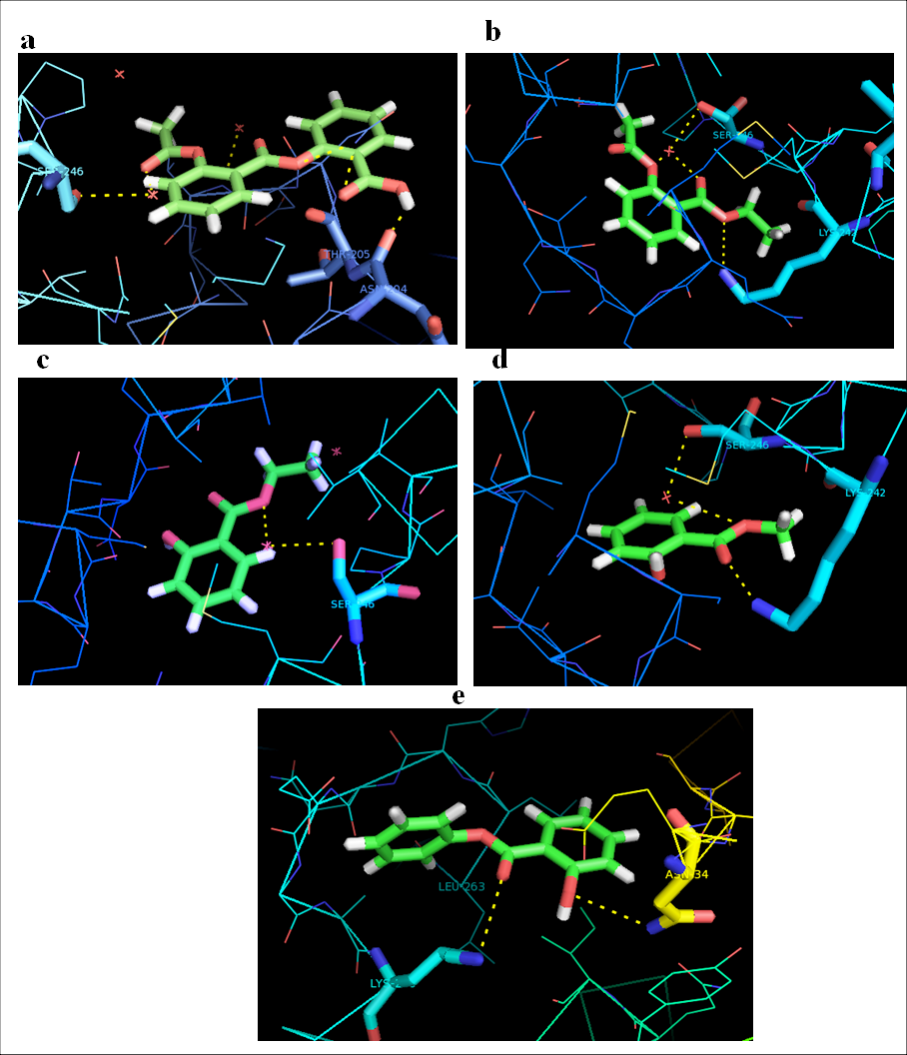
Interaction of β catenin with (a) Acetylsalicylsalicylic acid; (b) Ethylacetylsalicylate; (c) Ethylsalicylate; (d) Methylsalicylate; e) Phenylsalicylate

The molecular docking analysis data of aspirin analogues with β-catenin is given in Table 1 to 3 (see PDF). The interaction of aspirin analogues with β-catenin is given in Figure 1. The important amino acids residues present in the active site of protein were identified using MetaPocket 2.0 server. The predicted binding pocket comprises following amino acids ASN-204, THR-205, ASN-206, ASP-207, VAL- 208, LYS-242, SER-246, PRO- 247, VAL-248, LYS-263, LEU-264, LYS-508, GLU-568 & GLY- 572. Molecular docking studies Aspirin analogues with β-catenin were carried out based on the following parameters interacting amino acids, docking score and ACE values. Results of docking studies confirmed that most of interacting amino acids were present in the binding site through MetaPocket. The docking results of aspirin and its nine analogues were shown in Table 1 (see PDF). The atomic contact energy of (ACE) value of aspirin analogues ranges from -195.31 Kcal/mol to -76.36 Kcal/mol. The marketed FDA drug Aspirin showed the ACE value - -137.01 Kcal/mol, this shows that analogues of aspirin also showed the similar affinity towards the beta-catenin protein. Compared 9 analogues, the Acetylsalicylsalicylic acid showed the highest ACE value -195.31 Kcal/mol. In order to analysis the binding pattern of aspirin analogues based on the docking studies visual poses examination analysis has been carry out which had highest ACE value against the binding site of β-catenin taking into the account the occurrence of H-bond and their interaction key amino acids residues of the β-catenin in the binding site. Among them the amino acids residues ASN-204, SER -246,THR-205 plays a vital role in the mechanism of action of β-catenin protein. Mostly the amino acids residues LYS-242 & SER -246 alternatively form the H-bond interaction with target protein β-catenin. Hydrogen bond interactions of best five compounds were shown in Figure 1.

Absorption, Distribution, Metabolism and Excretion (ADME) is simple and essential analysis tool. Now days, it is usually accepted in the primary stage of drug development process, as of its exclusive feature nature. In the present, Drug-likeness properties of these five compounds was calculated using Lipinski rule of five and shown in Table 2 (see PDF). Results of ADME studies showed that selected compounds have good gastrointestinal (GI) absorption effect. Hence, the results of PatchDock and ADME analysis evidently proved that selected five analogues compounds have the ability to inhibit the β-catenin protein and act as potent anti cancer agents.

## Conclusion

We document the molecular docking analysis data of aspirin analogues with β-catenin for further consideration.
